# A case of prolonged cefiderocol therapy during continuous hemodiafiltration in a patient with an implanted left ventricular assist device

**DOI:** 10.1016/j.idcr.2026.e02725

**Published:** 2026-08-19

**Authors:** Hiroshi Kimura, Takaaki Yano, Yukiro Kurokawa, Shinobu Murakami, Hitoshi Miyamoto, Noriaki Hidaka, Hisamichi Tauchi, Mamoru Tanaka

**Affiliations:** a Division of Pharmacy, Ehime University Hospital, Toon, Ehime, Japan; b Division of Infection Control and Prevention, Ehime University Hospital, Toon, Ehime, Japan

**Keywords:** Cefiderocol, Continuous hemodiafiltration, Left ventricular assist device, *Pseudomonas aeruginosa*, Bacteremia

## Abstract

Cefiderocol (CFDC) is a siderophore cephalosporin with activity against multidrug-resistant Gram-negative pathogens, and it is considered a treatment option for refractory Gram-negative infections. Because CFDC is primarily eliminated by the kidneys, dose individualization is required in patients with renal impairment and in those receiving renal replacement therapy. During continuous hemodiafiltration (CHDF), drug clearance is influenced not only by residual renal function but also by dialysis and filtration flow rates and membrane characteristics; thus, careful determination of dose and dosing interval is necessary. However, real-world evidence on CFDC dosing during CHDF, particularly for prolonged courses, remains limited. We report a case of refractory *Pseudomonas aeruginosa* bacteremia in a patient with an implanted left ventricular assist device receiving CHDF, successfully managed with a 55-day course of CFDC using a dosing strategy tailored to CHDF settings.

## Highlights

• Prolonged cefiderocol (55 days) during CHDF in an LVAD recipient.
• Bacteremia cleared by Day 5 with dosing tailored to CHDF settings.
• Cefiderocol MIC 1 mg/L interpreted by CLSI M100 ED35 (2025).
• Pharmacist-led PK/PD dosing support enabled safe long-term therapy.
• No clear cefiderocol-related adverse events were observed.

## Introduction

Cefiderocol (CFDC) is a novel cephalosporin that utilizes a siderophore-mediated uptake mechanism and has activity against carbapenem-resistant Enterobacterales and multidrug-resistant *Pseudomonas aeruginosa*. Because CFDC is predominantly renally excreted, appropriate dose adjustment is required in patients with renal dysfunction and in those receiving renal replacement therapy [[1], [2], [3]].

In critically ill patients, continuous renal replacement therapy (CRRT), including continuous hemodiafiltration (CHDF), is frequently used as part of intensive care. Antibacterial clearance during CRRT is influenced by filtration and dialysate flow rates, treatment duration, residual renal function, and membrane properties. The U.S. Food and Drug Administration-approved prescribing information for cefiderocol provides initial dosing recommendations for patients receiving CRRT according to the effluent flow rate, while also noting that further dose individualization may be required according to residual renal function and the patient’s clinical status [4]. Wenzler et al. demonstrated that effluent flow rate is a major determinant of cefiderocol clearance during CRRT and supported the use of effluent flow rate-based regimens through pharmacokinetic/pharmacodynamic analyses [5]. Therefore, careful assessment of CRRT settings is required to avoid both inadequate exposure and drug accumulation.

Patients with an implanted left ventricular assist device (LVAD) are at high risk for infection due to the driveline and other prosthetic materials, and such infections can be difficult to eradicate when device exchange or removal is not feasible. Here, we describe a case of *P. aeruginosa* bacteremia in a patient with an LVAD receiving CHDF who was treated with CFDC for 55 days, with a focus on the dosing rationale and clinical course [6].

## Case presentation

**Fig. 1:**
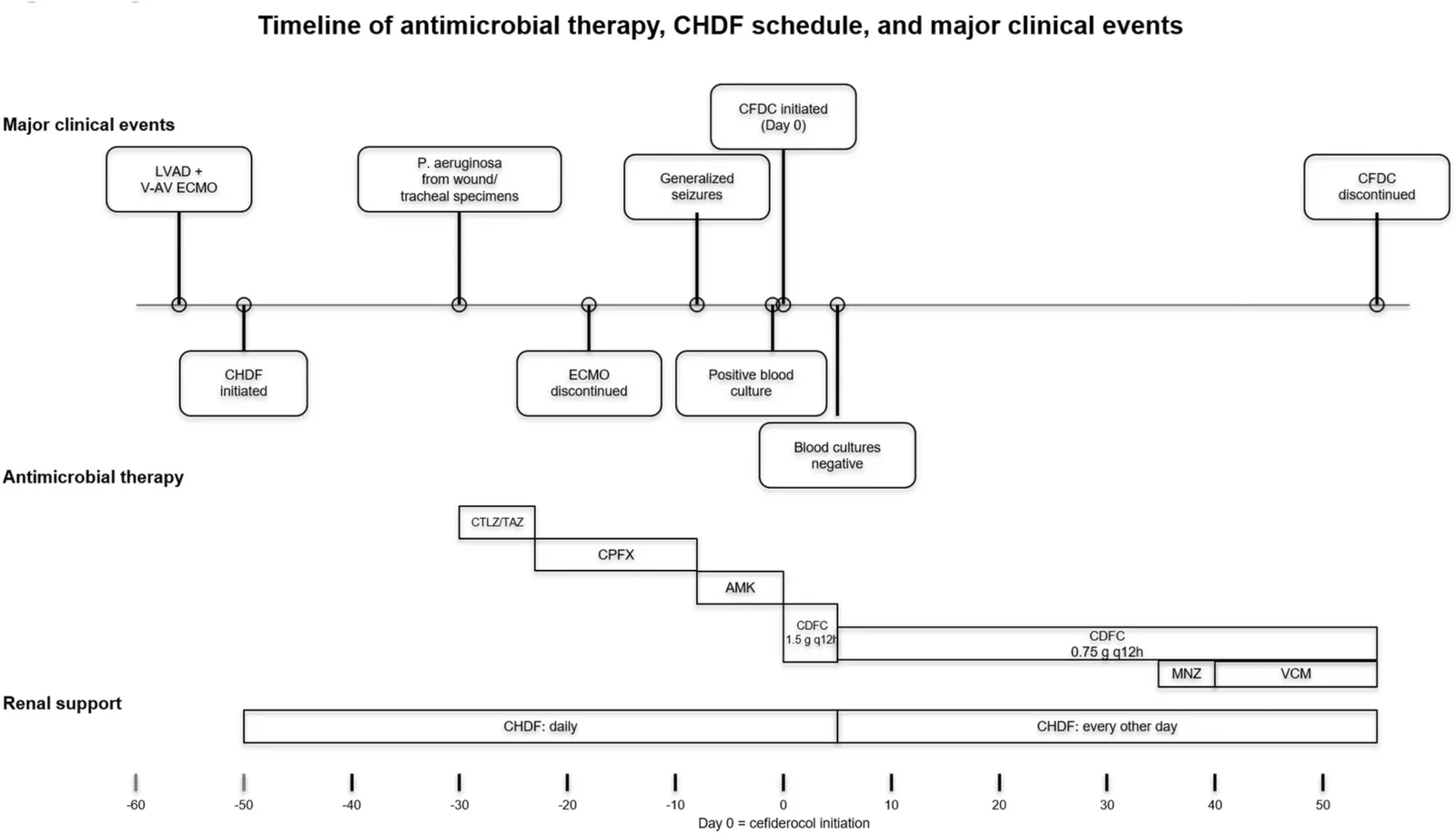
Timeline of antimicrobial therapy, CHDF schedule, microbiological findings, and major clinical events. Day 0 was defined as the day cefiderocol therapy was initiated. Negative day values indicate events occurring before cefiderocol initiation. AMK, amikacin; CFDC, cefiderocol; CHDF, continuous hemodiafiltration; CPFX, ciprofloxacin; CTLZ/TAZ, ceftolozane/tazobactam; MNZ, metronidazole; VCM, vancomycin; ECMO, extracorporeal membrane oxygenation; LVAD, left ventricular assist device; V-AV ECMO, veno-arterial-venous extracorporeal membrane oxygenation.

For clarity, all clinical events were expressed relative to cefiderocol initiation. Day 0 was defined as the day CFDC therapy was initiated, and negative day values indicate events occurring before CFDC initiation. A visual summary of antimicrobial therapy, the CHDF schedule, and major clinical events is provided in Fig. 1.

A woman in her 50 s had a history of bicuspid aortic valve with aortic stenosis, scar-related ventricular tachycardia, atrial flutter, and first-degree atrioventricular block. She had previously undergone aortic valve replacement and ascending aortic replacement. An implantable cardioverter-defibrillator with cardiac resynchronization therapy capability had been placed for ventricular tachycardia. She was admitted to a previous hospital after implantable cardioverter-defibrillator shocks and developed persistent circulatory failure with renal dysfunction, prompting transfer to our hospital for further management, including LVAD implantation.

On Day −56, an implantable LVAD and veno-arterial-venous extracorporeal membrane oxygenation were initiated. Because chest closure made blood pressure maintenance difficult, she was managed with an open chest. CHDF was started on Day −50. On Day −30, β-lactam-resistant P. aeruginosa was isolated from the open chest wound and tracheal secretions, and ceftolozane/tazobactam was initiated for the suspected wound and respiratory tract infection. On Day −23, treatment was changed to ciprofloxacin. The precise reason for this transition was not explicitly documented in the medical record; however, based on the treating physician’s assessment at that time, the change was considered to have been made because the clinical response to ceftolozane/tazobactam was insufficient. Extracorporeal membrane oxygenation was discontinued on Day −18. On Day −8, generalized seizures developed during ciprofloxacin therapy. Because a drug-related adverse effect could not be excluded, ciprofloxacin was discontinued, and amikacin was initiated based on the antimicrobial susceptibility results. On Day −1, blood cultures obtained during amikacin therapy grew P. aeruginosa. Despite in vitro susceptibility to amikacin, the occurrence of bacteremia during treatment suggested an inadequate clinical response. The antimicrobial stewardship team was therefore consulted, and cefiderocol was recommended and initiated on Day 0. The sequence of antipseudomonal therapy and the reasons for treatment transitions are summarized in Table 2.

**Table 1:** Antimicrobial susceptibility profile of the Pseudomonas aeruginosa bloodstream isolate obtained on Day −1.

Antimicrobial agent	Interpretation	MIC（mg/L）
Ampicillin		> 16
Ampicillin/sulbactam		> 16
Piperacillin/tazobactam	I	32
Cefcapene		> 4
Cefazolin		> 32
Ceftriaxone		> 32
Sulbactam/cefoperazone	I	32
Ceftazidime	R	> 32
Cefepime	I	16
Cefmetazole		> 32
Flomoxef		> 32
Ceftolozane/tazobactam	R	NA
Imipenem	R	NA
Meropenem	R	> 8
Doripenem	R	8
Gentamicin	S	≤ 0.5
Amikacin	S	1
Arbekacin	S	NA
Minocycline		> 8
Levofloxacin	R	> 4
Ciprofloxacin	R	4
Fosfomycin	S	32
Cefiderocol	S	1

Note: The susceptibility profile shown represents the bloodstream isolate obtained on Day −1 during amikacin therapy. Interpretation (S/I/R) for cefiderocol MIC was based on CLSI M100 ED35 [7]. NA, not available.

**Table 2:** Antipseudomonal antimicrobial therapy and reasons for treatment transitions.

**Antimicrobial agent**	**Treatment period**	**Indication or basis for selection**	**Reason for discontinuation or transition**
Ceftolozane/tazobactam	Day −30 to Day −23	β-lactam-resistant P. aeruginosa isolated from the open chest wound and tracheal secretions	The precise reason was not explicitly documented; based on the treating physician’s assessment, an insufficient clinical response was considered the reason for the transition.
Ciprofloxacin	Day −23 to Day −8	Alternative antipseudomonal therapy after discontinuation of ceftolozane/tazobactam	Generalized seizures developed; a drug-related adverse effect could not be excluded.
Amikacin	Day −8 to Day 0	Selected based on in vitro susceptibility	P. aeruginosa bacteremia developed during therapy, suggesting an inadequate clinical response.
Cefiderocol	Day 0 to Day 55	Multidrug-resistant bloodstream isolate; CFDC MIC 1 mg/L and susceptible according to CLSI M100 ED35	Treatment completed after sustained clinical and microbiological improvement.

Abbreviations: CFDC, cefiderocol; CLSI, Clinical and Laboratory Standards Institute; MIC, minimum inhibitory concentration.

Antimicrobial susceptibility testing of the P. aeruginosa bloodstream isolate obtained on Day −1 showed resistance to ceftolozane/tazobactam, ceftazidime, and carbapenems; intermediate susceptibility to piperacillin/tazobactam and cefepime; and susceptibility to aminoglycosides. The CFDC minimum inhibitory concentration was 1 mg/L and was interpreted as susceptible according to Clinical and Laboratory Standards Institute (CLSI) M100, 35th edition [7] (Table 1).

Blood cultures became negative on Day 5 after starting CFDC, and no subsequent blood cultures became positive. Because prosthetic material-associated infection was suspected and exchange or removal of the LVAD was not feasible, prolonged antimicrobial therapy was considered necessary. CFDC was continued through Day 55, for a total duration of 55 days. Treatment was discontinued after sustained blood culture negativity, continued improvement in fever and wound findings, and the absence of clinical findings suggestive of relapse.

## CFDC dosing strategy

A clinical pharmacist proposed the initial CFDC dose and subsequent dose adjustment based on the CHDF settings, the FDA-approved U.S. prescribing information, and the available pharmacokinetic evidence for cefiderocol during CRRT [4,5].

### CHDF settings

• Days 1–4 (daily): blood flow 150 mL/min; filtration flow 0.9 L/h; dialysate flow 0.6 L/h; replacement fluid 0.2 L/h; filter: AN69 membrane.
• Day 5 onward (every other day): blood flow 150 mL/min; filtration flow 1.03 L/h; dialysate flow 0.8 L/h; replacement fluid 0.2 L/h; filter: AN69 membrane.

### CFDC regimen

• Days 0–4: CFDC 1.5 g every 12 h, infused over 3 h.
• The combined filtration and dialysate flow rate during daily CHDF was approximately 1.5 L/h; therefore, the initial regimen of 1.5 g every 12 h was consistent with the FDA-approved recommendation for patients receiving CRRT with an effluent flow rate of ≤ 2 L/h [4].
• Day 5 onward: CFDC 0.75 g every 12 h, infused over 3 h (dose reduction after the change in CHDF frequency).

After CHDF frequency was reduced from daily to every other day, the combined filtration and dialysate flow rate on CHDF days was approximately 1.83 L/h, whereas extracorporeal clearance was absent on non-CHDF days. The treating physicians, clinical pharmacists, and clinical engineers discussed the CHDF schedule and the patient’s residual renal function, which was clinically estimated to correspond to a glomerular filtration rate of approximately 15–20 mL/min/1.73 m². Because the FDA-approved prescribing information does not provide a specific regimen for alternate-day CHDF, the dose of 0.75 g every 12 h was selected as an individualized regimen after multidisciplinary consideration of the intermittent extracorporeal clearance, estimated residual renal function, and potential risk of drug accumulation. This dose was not an FDA-approved regimen for CRRT and was not directly derived from a single FDA renal-function category. Therapeutic drug monitoring of cefiderocol was not performed because a clinically available validated assay was not available; therefore, adequate drug exposure could not be directly confirmed by measured concentrations. The dose adjustment was based on the change in CHDF frequency and the anticipated reduction in overall extracorporeal clearance, with the aim of balancing the risks of underexposure and accumulation.

## Effectiveness

After initiation of CFDC, inflammatory markers improved; C-reactive protein decreased from 15.33 mg/dL at initiation to 5.41 mg/dL on Day 2. Blood cultures were negative for *P. aeruginosa* on Day 5. Although prolonged therapy was required because of presumed prosthesis-associated infection, both clinical and microbiological improvement were observed.

## Safety

**Fig. 2:**
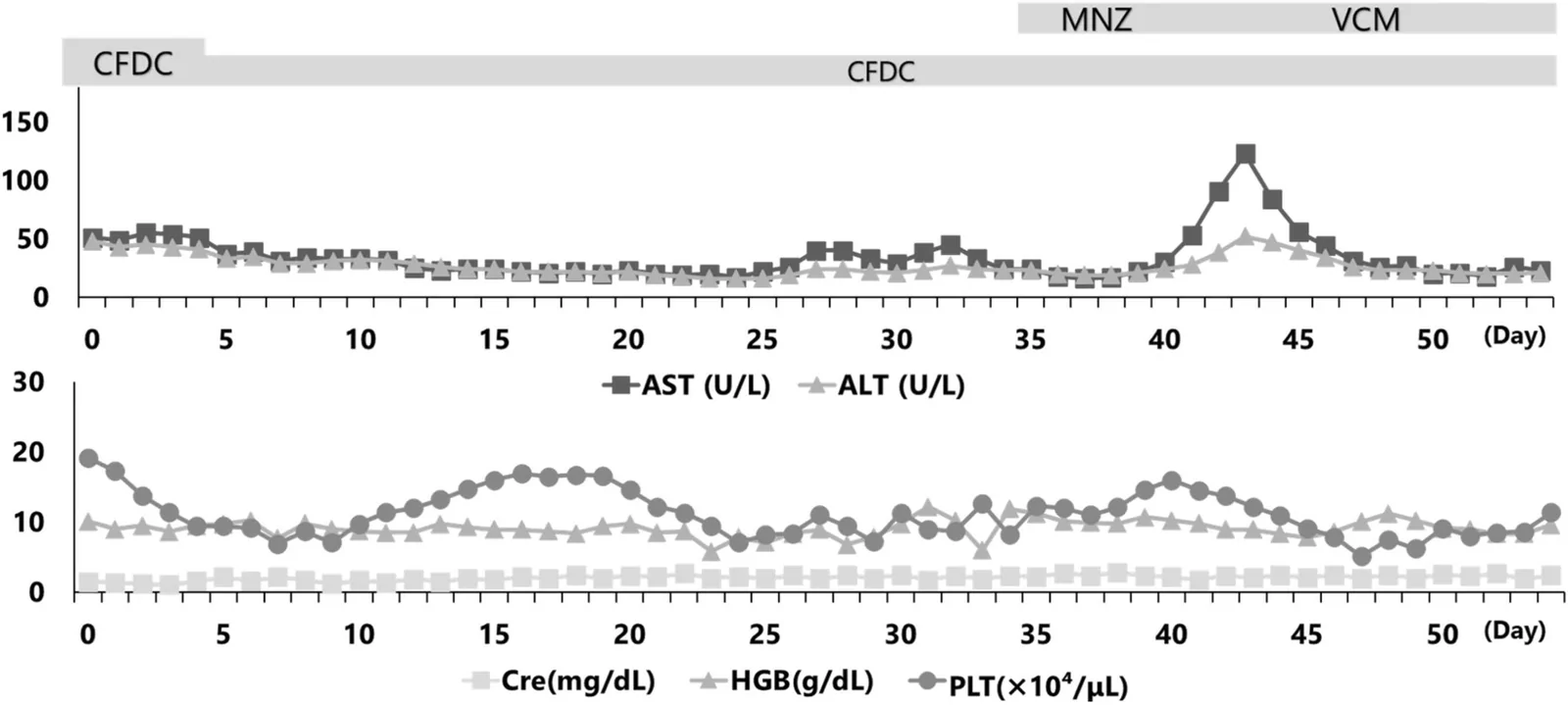
Clinical course during cefiderocol therapy, including time trends in AST, ALT, serum creatinine, hemoglobin, and platelet count. ALT, alanine aminotransferase; AST, aspartate aminotransferase; CFDC, cefiderocol; Cr, serum creatinine; HGB, hemoglobin; MNZ, metronidazole; PLT, platelet count; VCM, vancomycin.

Fig. 2 shows the time course of aspartate aminotransferase (AST), alanine aminotransferase(ALT), serum creatinine, hemoglobin, and platelet count. No clear CFDC-related adverse events were observed during therapy. An increase in AST around Day 40 was considered attributable to metronidazole, and AST promptly decreased after its discontinuation. Given the patient’s critical illness and multiple concomitant medications, assessment of adverse events took into account underlying conditions and co-therapies.

Vancomycin and metronidazole were empirically added during portions of the cefiderocol treatment period because a concomitant infection caused by organisms other than P. aeruginosa was suspected. However, no apparent clinical improvement was observed, and both agents were subsequently discontinued. Neither agent was administered for P. aeruginosa bacteremia, and no additional antipseudomonal antimicrobial agent was administered concurrently with cefiderocol. Thus, cefiderocol was used as antipseudomonal monotherapy.

## Discussion

This case describes prolonged CFDC therapy for refractory *P. aeruginosa* infection in a patient with an implanted LVAD receiving CHDF. In LVAD recipients, device-related and wound infections can be protracted, and intensive care management can cause marked changes in fluid status, organ perfusion, and inflammatory state, potentially destabilizing antibiotic pharmacokinetics. Therefore, appropriate antibiotic selection and dosing may substantially affect outcomes.

Because CFDC is primarily eliminated by the kidneys, appropriate dosing is particularly important in patients receiving renal replacement therapy. The FDA-approved prescribing information provides initial CRRT dosing recommendations according to effluent flow rate: 1.5 g every 12 h for an effluent flow rate of ≤ 2 L/h, with higher doses or shorter dosing intervals recommended as the effluent flow rate increases [4]. These recommendations are intended as initial regimens and may require modification according to residual renal function and the patient’s clinical status. Wenzler et al. identified effluent flow rate as a major determinant of cefiderocol clearance during CRRT and demonstrated through pharmacokinetic/pharmacodynamic modeling that effluent flow rate-based regimens were capable of achieving the relevant pharmacodynamic targets [5]. Nevertheless, clinical experience with prolonged cefiderocol administration during CHDF remains limited, particularly when the frequency of CHDF changes during treatment.

In this case, several factors coexisted: CHDF, LVAD management, severe infection, and prolonged treatment. These factors may influence not only pharmacokinetics but also source-control feasibility and clinical response; thus, dosing adjustments based solely on renal function may be insufficient. The observed microbiological clearance with continued CFDC supports its potential usefulness in refractory *P. aeruginosa* infection during CHDF.

The findings in this case should be interpreted with caution. As a single case report, therapeutic success cannot be attributed to CFDC alone. Improvements in overall condition, device management, infection control measures, and supportive care may also have contributed. Moreover, pharmacokinetics may have changed over time with CHDF and the patient’s clinical status. Therapeutic drug monitoring and pharmacokinetic/pharmacodynamic (PK/PD)-based evaluation would allow more precise assessment. Accumulation of additional cases and PK analyses across CHDF settings is needed to refine optimal dosing.

The dose of 0.75 g every 12 h used after Day 5 was selected through multidisciplinary discussion among the treating physicians, clinical pharmacists, and clinical engineers, taking into account alternate-day CHDF and residual renal function clinically estimated to correspond to a glomerular filtration rate of approximately 15–20 mL/min/1.73 m². However, this regimen is not included in the FDA-approved CRRT dosing recommendations and was not directly derived from a single FDA renal-function category. Residual renal clearance was not formally measured, and cefiderocol concentrations were not determined. Therefore, neither the independent contribution of residual renal function to cefiderocol clearance nor attainment of the pharmacokinetic/pharmacodynamic target during the reduced-dose period could be precisely confirmed. Although blood cultures became negative and no microbiological relapse was observed during therapy, these clinical findings cannot substitute for pharmacokinetic confirmation.

The optimal duration of antimicrobial therapy for LVAD-associated infection has not been firmly established and depends on the depth and extent of infection, the causative organism, and the feasibility of source control. Consensus recommendations for mechanical circulatory support device-related infection support prolonged targeted antimicrobial therapy, generally for approximately 4–6 weeks, and emphasize individualized management when the infected device cannot be removed or exchanged [8,9]. In the present case, the LVAD could not be exchanged, and the preceding P. aeruginosa bacteremia raised concern for persistent infection involving prosthetic material. Consequently, CFDC was continued for 55 days. This duration was individualized according to the sustained microbiological response, continued improvement in fever and wound findings, and the absence of clinical evidence of relapse, and should not be interpreted as an established standard duration for cefiderocol therapy.

A key aspect of novelty is the ability to continue CFDC for a relatively prolonged period in a patient with both LVAD support and CHDF—a setting with high complexity in both infection control and pharmacokinetics. Published reports on CFDC during CRRT have used heterogeneous doses, intervals, and infusion durations, and standardized, setting-based guidance incorporating effluent dose and membrane characteristics remains insufficient. Our case provides practical insight into tailoring CFDC dosing to CHDF conditions in a critically ill patient with an LVAD [[4], [5], [6],[10], [11], [12], [13]].

Finally, this case highlights the importance of viewing CFDC therapy not merely as drug selection but as part of a comprehensive infection-management strategy that incorporates CHDF settings, longitudinal clinical evaluation, and microbiological monitoring. Pharmacist-led dosing support with PK/PD considerations may be clinically beneficial in similar refractory, device-associated infections during CHDF.

## CRediT authorship contribution statement

**Hiroshi Kimura:** Conceptualization, Data curation, Investigation, Writing – original draft. **Takaaki Yano:** Investigation, Writing – review & editing. **Yukiro Kurokawa:** Investigation, Writing – review & editing. **Shinobu Murakami:** Data curation, Visualization, Writing – review & editing. **Hitoshi Miyamoto:** Data curation, Visualization, Writing – review & editing. **Noriaki Hidaka:** Supervision, Writing – review & editing. **Hisamichi Tauchi:** Investigation, Supervision, Writing – review & editing. **Mamoru Tanaka:** Supervision, Writing – review & editing.

## Ethics declaration

Written informed consent to take part in the study and to publish the article has been obtained from all participants or their legal representatives. The privacy rights of participants have been observed.

This study was performed in compliance with relevant laws, regulatory frameworks and guidelines where the research took place. Ethics committee approval was not required under relevant laws and institutional guidelines. Ethics approval was not required for this case report in accordance with the institutional policy of Ehime University Hospital. The case was prepared in compliance with relevant institutional guidelines and with due consideration for the patient’s rights, privacy, and confidentiality. Written informed consent for publication was obtained from the patient’s legally authorized representative.

This research follows the CARE guidelines and the CARE checklist.

## Declaration of Generative AI and AI-assisted technologies in the writing process

Generative AI was used to assist with language editing and polishing during manuscript preparation. The authors take full responsibility for the accuracy and integrity of the content.

## Funding

This research did not receive any specific grant from funding agencies in the public, commercial, or not-for-profit sectors.

## Declaration of Competing Interest

The authors declare that they have no known competing financial interests or personal relationships that could have appeared to influence the work reported in this paper.
